# BARD1 mediates TGF-β signaling in pulmonary fibrosis

**DOI:** 10.1186/s12931-015-0278-3

**Published:** 2015-09-29

**Authors:** Pierre-Alain André, Cecilia M. Prêle, Sarah Vierkotten, Stéphanie Carnesecchi, Yves Donati, Rachel C. Chambers, Jean-Claude Pache, Bruno Crestani, Constance Barazzone-Argiroffo, Melanie Königshoff, Geoffrey J. Laurent, Irmgard Irminger-Finger

**Affiliations:** Molecular Gynecology and Obstetrics Laboratory, Department of Gynecology and Obstetrics, Geneva University Hospitals, Geneva, Switzerland; Department of Genetic and Laboratory Medicine, Geneva University Hospitals, Geneva, Switzerland; Institute for Respiratory Health, University of Western Australia, Nedlands, WA Australia; Centre for Cell Therapy and Regenerative Medicine, School of Medicine and Pharmacology, University of Western Australia, Harry Perkins Institute of Medical Research, Nedlands, WA Australia; Comprehensive Pneumology Center Ludwig Maximilians University, University Hospital Grosshadern and Helmholtz Zentrum München, Munich, Germany; Department of Pediatrics and Pathology/Immunology, University of Geneva, Geneva, Switzerland; Centre for Inflammation and Tissue Repair, University College London, London, UK; Department of Clinical Pathology, Geneva University Hospitals, Geneva, Switzerland; INSERM, Unité 1152, University of Paris Diderot and Hopital Bichat, Paris, France

**Keywords:** BARD1, TGF-β, Lung fibrosis, Apoptosis, Proliferation, Differential splicing, Epithelial-mesenchymal transition (EMT)

## Abstract

**Background:**

Idiopathic pulmonary fibrosis (IPF) is a rapid progressive fibro-proliferative disorder with poor prognosis similar to lung cancer. The pathogenesis of IPF is uncertain, but loss of epithelial cells and fibroblast proliferation are thought to be central processes. Previous reports have shown that *BARD1* expression is upregulated in response to hypoxia and associated with TGF-β signaling, both recognized factors driving lung fibrosis. Differentially spliced BARD1 isoforms, in particular BARD1β, are oncogenic drivers of proliferation in cancers of various origins. We therefore hypothesized that BARD1 and/or its isoforms might play a role in lung fibrosis.

**Methods:**

We investigated *BARD1* expression as a function of TGF-β in cultured cells, in mice with experimentally induced lung fibrosis, and in lung biopsies from pulmonary fibrosis patients.

**Results:**

FL BARD1 and BARD1β were upregulated in response to TGF-β in epithelial cells and fibroblasts *in vitro* and *in vivo*. Protein and mRNA expression studies showed very low expression in healthy lung tissues, but upregulated expression of full length (FL) BARD1 and BARD1β in fibrotic tissues.

**Conclusion:**

Our data suggest that FL BARD1 and BARD1β might be mediators of pleiotropic effects of TGF-β. In particular BARD1β might be a driver of proliferation and of pulmonary fibrosis pathogenesis and progression and represent a target for treatment.

**Electronic supplementary material:**

The online version of this article (doi:10.1186/s12931-015-0278-3) contains supplementary material, which is available to authorized users.

## Introduction

Idiopathic Pulmonary Fibrosis (IPF) is a chronic, progressive and fatal disease with limited treatment options [1, 2]. Of the main subtypes of IPF, usual interstitial pneumonia (UIP) is typically associated with extensive and persistent lung fibrosis, and non-specific interstitial pneumonia (NSIP) is associated with inflammation and fibrosis. The incidence of these diseases is increasing and is reported to be about 20 per 100,000 [3, 4].

The pathogenesis of IPF is poorly understood, but is thought to be caused by epithelial damage followed by a chronic progression of the healing response, resulting in excessive deposition of extracellular matrix proteins without resolution [5, 6]. Transforming Growth Factor-β (TGF-β) is generated via multiple molecular pathways associated with lung fibrosis [1, 7] and is one of a number of factors known to drive fibrosis. TGF-β signals through Smad3 to induce collagen production, a hallmark of lung fibrosis [8, 9]. Many inflammatory cells, central to fibrosis, store and secrete TGF-β. Coagulation cascade proteins, such as thrombin, are known to induce TGF-β activation [10], as does oxidative stress [11]. TGF-β is thought to drive epithelial cell apoptosis, epithelial-mesenchymal transition (EMT), fibroblast proliferation, and collagen deposition, key processes to the initiation and progression of lung fibrosis.

Currently, there are no molecular biomarkers in widespread clinical use for IPF. Several susceptibility loci have been identified by genome wide association studies [12], but they have not been validated as biomarkers or drug targets as yet.

We hypothesized that BARD1 (BRCA1-associated RING domain 1), which is aberrantly expressed and correlated with poor prognosis in lung cancer [13], might be involved in lung fibrosis. BARD1 is a major binding protein of the breast cancer predisposition gene product BRCA1 and a tumor suppressor in its own right [14]. Bound to BRCA1, BARD1 is essential for BRCA1’s E3 ubiquitin ligase and tumor suppressor activity in DNA repair and cell cycle control [15]. Independently of BRCA1, BARD1 is an inducer of apoptosis by binding to and stabilizing p53 [16–18] and is upregulated in response to hormones, genotoxic stress, and hypoxia [16, 18–20]. However, BARD1 expression is also upregulated in proliferative tissues [21], where it has essential functions in mitosis [17, 22, 23]. Thus, there is strong evidence that BARD1 is involved in apoptosis and in the control of proliferation.

Alternatively spliced isoforms of BARD1 have been identified in various cancers correlated with disease progression and poor prognosis [13, 24–28]. These isoforms, abundantly expressed in tumors and cancer cell lines, lack the BRCA1-interacting N-terminal RING domain and do not retain tumor suppressor functions. In particular BARD1β has been shown to be essential for cell proliferation and for promoting fibroblast proliferation [23, 25]. BARD1β is considered a driver of tumorigenesis, as its expression correlates with tumor progression, poor prognosis, and decreased survival time of lung cancer patients [13].

The role of BARD1 in the development and progression of proliferative lung diseases other than cancer remains unknown. Recent reports suggest that the pathogenesis of lung cancer and lung fibrosis share common features [29], and, for example, persistent epithelial cell damage and enhanced fibroblast proliferation are observed in both diseases, with IPF patients showing an 8–14 fold increase in lung cancer incidence [30].

Hallmarks of lung fibrosis are thought to be hypoxia, loss of epithelial cells due to apoptosis and/or EMT, and fibroblast proliferation [31, 32]. BARD1 was described as a mediator of stress signals, including hypoxia, towards apoptosis *in vitro* and *in vivo* [16]. BARD1 might also be linked to TGF-β signaling, as upregulated *BARD1* expression was shown along with TGF-β early response genes in breast cancer [33] and was associated with endoglin upregulation, a co-receptor for TGF-β [34].

Based on the key properties of BARD1 and its isoforms, and the observed features that characterize pulmonary fibrosis, we hypothesized that full length (FL) BARD1 and/or its isoforms might play a role in this disease. To test this hypothesis, we investigated the possible mechanisms of BARD1 actions by exploring the regulation of FL BARD1 and isoform expression by TGF-β and by their exogenous overexpression *in vitro*. We also analyzed BARD1 expression in lung tissues from patients with IPF and those of mice following experimentally induced lung fibrosis.

### Materials and methods

#### Clinical data

Human lung biopsies of patients with Idiopathic Interstitial Pneumonias (IIP), IPF and NSIP, and human control lung tissue samples, were obtained in accordance to an approved protocol by the Institutional Ethics Committees of the University Hospitals Geneva (HUG) and the Hôpital Bichat, Paris. In total, 17 IIP samples and seven control lung tissue samples were analyzed. Control lung tissue samples were obtained from resected lung tissue collected during lung cancer surgery; samples were taken at sites distal to tumors. Five control samples were obtained from patients with other lung diseases, which included emphysema, tuberculosis, and carcinoid dysplasia. All patients were informed and approvals were obtained from the local ethics committees.

Paraffin-embedded sections from human tissue biopsies and air-inflated, perfusion-fixed lungs from mice were immune stained for BARD1 and α-smooth-muscle-actin (α-SMA). The primary antibodies used in the study were: BARD1-N19 (sc-7373; Santa Cruz; dilution 1:25) (BARD1 exon 1); BARD1-C20 (sc-7372; Santa Cruz; dilution 1:20) (BARD1 exon 11); BARD1-BL (A300-263A; Bethyl Laboratories; dilution 1:50) (middle exon 4); α-smooth muscle actin (ab5694; Abcam, dilution 1:100); BARD1-p25 (directed against sequence of alternative open reading frame of BARD1β) [23, 25] (dilution 1:50); p53 (sc-6243 Santa Cruz, dilution 1:20); Bax (sc-493; Santa Cruz; dilution 1:100). Secondary, horseradish-conjugated rabbit or goat antibodies were used at a 1:100 dilution. Then diaminobenzidine DAB staining was performed during 15 min at room temperature. Slides were counterstained with haematoxylin.

#### Mouse model of bleomycin-induced pulmonary fibrosis

C57BL/6J male mice aged 12–16 weeks were kept in specific pathogen-free conditions. All animal experiments were approved by the Institutional Ethics Committee of Animal Care in Geneva and the Cantonal Veterinary Office. Male mice, weighing 20–30 g, were anesthetized with ketamine/xylazine (i.p., 90/3.8 mg/kg). Bleomycin or saline was instilled intratracheally at a dose of 2U/kg to mice using an endotracheal catheter attached to a 50-ml Hamilton syringe (Hamilton Bonaduz) as previously described [35]. Mice were killed at day 3 or 15 after bleomycin treatment and lungs were processed for subsequent analysis.

#### Cell culture

The normal human fibroblasts CCD-19Lu, bronchial epithelial cells 16HBE and NuLi-1, alveolar basal epithelial cells A549, and mouse fibroblasts L929 were cultured in RPMI 1640 Medium, GlutaMAX™ (Invitrogen), 10 % fetal calf serum (FCS), 200U/ml penicillin and 200U/ml streptomycin at 37 °C in a 5 % of CO_2_ incubator. For TGF-β1 treatment, cells were serum starved in 0.5 % FCS overnight prior to stimulation with 10 ng/ml recombinant TGF-β1 (Biolegend) for 24 or 48 h. In the Smad signaling inhibition assay, SB431542 (Abcam) was added to the media at a concentration of 10 μM 1 h prior TGF-β1. Following stimulation cell lysates were prepared for RNA extraction, using Qiagen RNAeasy kit (Qiagen), for protein extraction or cells were fixed for immunofluorescence assays. Details are available in the online supplement.

#### Statistical analysis

Results are expressed as mean-standard deviation as indicated and were analyzed with the two-tailed Student’s *t* test (GraphPad Prism). Significance level was set at *p* < 0.05.

## Results

### FL BARD1 and BARD1β expression changes in response to TGF-β

**Fig. 1:**
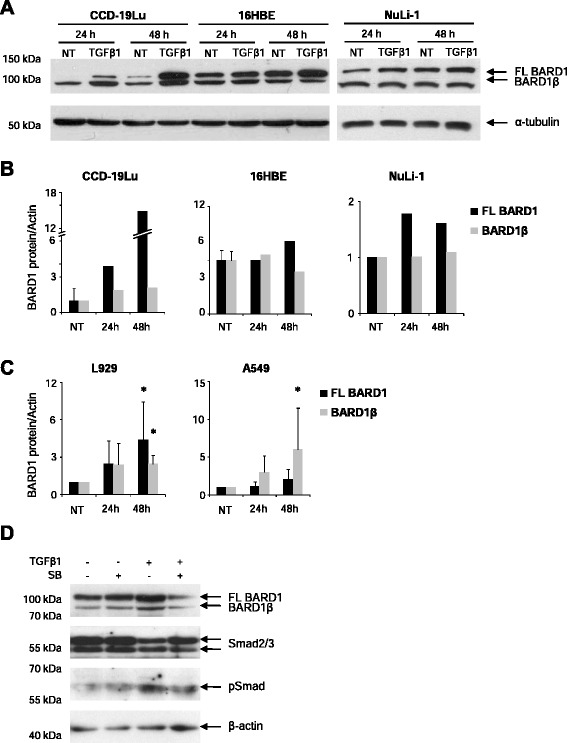
TGF-β modulates BARD1 expression. Human lung fibroblasts (CCD-19Lu), bronchial epithelial cells (16HBE, NuLi-1), alveolar basal epithelial cells (A549), and mouse fibroblasts (L929) were cultured in absence (NT) or presence of TGF-β1 (10 ng/ml), and cell extracts were prepared after 24, and 48 h of treatment. **a** Western Blots of CCD-19Lu, 16HBE and NuLi-1 cell extracts prepared at 24 and 48 h of TGF-β treatment, probed with anti-BARD1 BL (mapping exon 4 of BARD1) antibody recognizing FL BARD1 and BARD1β. **b** CCD19-Lu and 16HBE and (**c**) A549 and L929 Western Blots were quantified by measuring spot density using AlphaEaseFc software. Each bar represents mean + SD (*n* ≥2 experiments); two-tailed Student’s *T*-Test *p < 0.05, TGF-β1 versus non-treated. **d** A549 culture in presence of SB-431542 1 h prior TGF-β1 treatment. Cell extracts were prepared after 48 h of TGF-β1/SB-431542 treatment and probed with anti-BARD1 BL, anti-Smad2/3, anti-pSmad antibodies

As BARD1 was shown to be induced along with TGF-β early response genes [33] and to be regulated by the TGF-β co-receptor endoglin [34], we investigated the relationship between TGF-β and BARD1 expression *in vitro*. We incubated epithelial cells and fibroblasts with TGF-β and evaluated expression changes of BARD1 and/or its isoforms in response to TGF-β (Fig. 1).

After TGF-β treatment of CCD-19Lu lung fibroblasts, the expression of FL BARD1, a protein of 95 kD, as well as BARD1β, 82 kD, was upregulated after 24 and 48 h (Fig. 1a, b). We also used bronchial epithelial cells 16HBE and NuLi-1 to monitor BARD1 expression after TGF-β treatment. In these epithelial cells the increase in BARD1 expression in response to TGF-β was only moderate, and FL BARD1 expression increased, but less so BARD1β. To investigate whether BARD1 upregulation in response to TGF-β was observed in other cell lines we also tested L929 fibroblasts and A549 lung epithelial cells (Fig. 1c), as well as LL86 and HS888, normal lung fibroblasts and HeLa and HEK 293, epithelial cells (data not shown). FL BARD1 and BARD1β were upregulated in L929 cells with a similar pattern as observed for CCD-19Lu fibroblasts. The response in A549 cells was different from 16HBE and NuLI-1 epithelial cells, and a higher increase of BARD1β expression was observed (Fig. 1c). All other cell lines showed an increase at 24 h, but great variability after 48 h. To demonstrate that BARD1 expression was induced directly by TGF-β, we used a SMAD inhibitor and monitored BARD1 expression in response to TGF-β (Fig. 1d). In the presence of the SMAD inhibitor TGF-β-dependent FL BARD1 and BARD1β upregulation was reduced to levels without TGF-β stimulation.

As hypoxia is thought to also act as driver of lung fibrosis, we investigated whether BARD1 expression was modified by hypoxia in lung fibrosis. Both FL BARD1 and BARD1β were upregulated in lung epithelial cells and fibroblasts upon hypoxic treatment (Additional file 1: Figure S1). Therefore both factors hypoxia and TGF-β are activators of BARD1 expression.

**Fig. 2:**
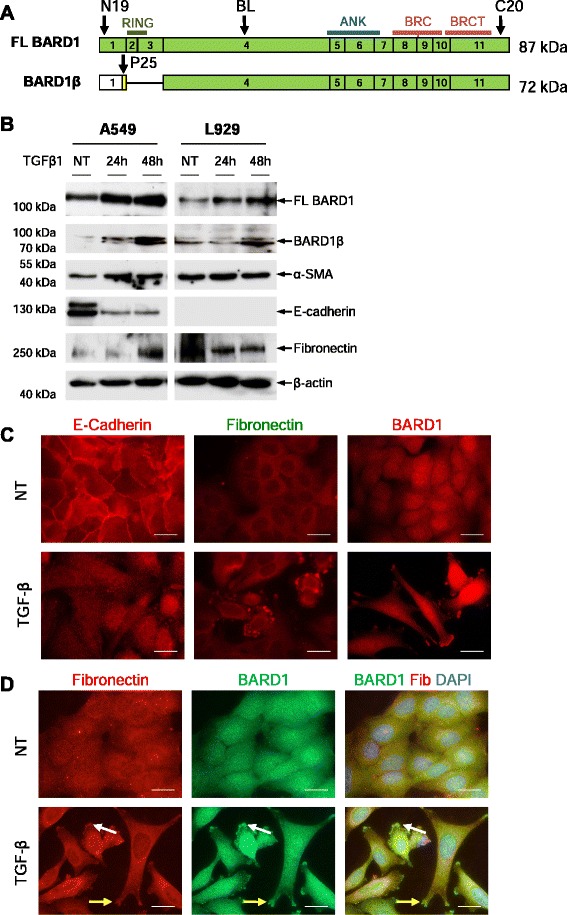
TGF-β induces BARD1β overexpression and localization. **a** Schematic representation of cDNA structure of FL BARD1 and N-terminally truncated isoform BARD1β. Approximate locations of protein motifs RING finger (RING), ankyrin repeats (ANK), and BRCT domains (BRCT) of BARD1 are indicated. Green exons indicate protein coding open reading frame (ORF). BARD1β encodes an alternative ORF in the first exon. Arrows indicated approximate positions of epitopes reactive with the antibodies used. **b** Lung epithelial cells (A549) and fibroblasts (L929) were cultured in absence (NT) or presence of TGF-β1, and cell extracts were prepared after 24 and 48 h. Western blots of cell extracts were probed with anti-BARD1 BL (exon 4), BARD1β-specific P25 (alternative ORF of BARD1β), α-SMA, E-cadherin, fibronectin, and β-actin antibodies. **c** A549 were treated with TGF-β1 and cells were fixed after 48 h. Immunofluoresence was performed with anti-E-cadherin, fibronectin, or BARD1 BL (exon 4) antibodies. **d** Co-immunostaining with anti-BARD1 BL and fibronectin (Fib) antibodies. TGF-β1 modulates BARD1 localization to areas at the cell membrane (*yellow arrows*) and cytoplasmic vesicles (*white arrows*), similar to and co-staining with fibronectin. Scale bars 20 μm

We have shown previously that BARD1β promotes cancer cell proliferation and induces proliferation of non-transformed fibroblasts [23, 25], we wanted to monitor the specific expression of FL BARD1 and BARD1β in response to TGF-β treatment. We probed Western blots of A549 and L929 cells cultured in the absence or presence of TGF-β for expression of FL BARD1 and BARD1β using an antibody specifically recognizing the N-terminal region of BARD1β, P25 [23, 25] (Fig. 2a). A549 cells and L929, both showed an increase of FL BARD1 expression, as well as an upregulation of BARD1β after treatment with TGF-β. The increase of FL BARD1 and BARD1β expression in A549 cells was accompanied by an increase of α-smooth muscle actin and fibronectin, as well as the decrease of E-cadherin, consistent with the well-described function of TGF-β as EMT inducer [36, 37] (Fig. 2b).

We performed immunofluorescence microscopy to assess the subcellular distribution of BARD1 upon TGF-β treatment. We observed that BARD1 expression and localization changed in parallel to changes in expression of markers of EMT (Fig. 2c). In non-treated cells, although under starving conditions, E-cadherin was observed both at the cell-cell adhesion sites and close to the nuclear membrane. Fibronectin showed nuclear and cytoplasmic localization. BARD1 expression was observed mostly in the nucleus and less in the cytoplasm, and not at cell adhesion sites. In TGF-β treated cells, E-cadherin staining was lost at cell adhesion sites and appeared diffuse in the nucleus and cytoplasm and intense staining was observed in the nucleus in approximately 10 percent of cells. Fibronectin expression was increased with TGF-β treatment and localized to the nuclear membrane and to vesicles at the cell membrane, presumably cell adhesion points. As observed on Western blots, BARD1 staining was generally stronger in the TGF-β treated cells and localized to the nucleus and cytoplasm (Fig. 2c). Specifically elevated BARD1 expression was observed in dots at the cell membrane, as observed for fibronectin staining. Double labeling for fibronectin and BARD1 showed co-localization in the cytoplasm and in particular in the presumed cell adhesion points (Fig. 2d).

Elevated FL BARD1 and BARD1β expression in parallel to fibronectin upregulation and E-cadherin repression, both features of EMT, suggested that BARD1 played a role in this pathway. To determine whether FL BARD1 or BARD1β play a role in TGF-β-mediated EMT induction, we exogenously expressed FL BARD1 or BARD1β in A549 cells and monitored their expression, and the effect thereof on fibronectin and E-cadherin expression and localization.

**Fig. 3:**
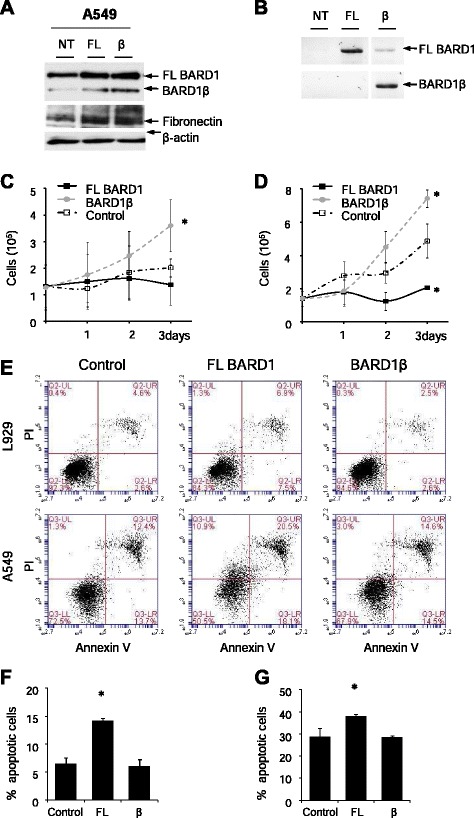
Differential effect of FL BARD1 and BARD1β on cell proliferation and apoptosis. **a**-**b** A549 cells were transfected with FL BARD1 and BARD1β expressing plasmids and cell extracts were prepared after 48 h. **a** Western Blots probed with anti-BARD1 (BL) and fibronectin. **b** RT-PCR analysis performed with primers for amplification of full length (FL) BARD1 (exon 1 to exon 11) or isoform BARD1β using primers from exon 1/4 junction and exon 11. **c** A549 and (**d**) L929 cell proliferation of control transformed cells (pcDNA), and cells expressing exogenous FL BARD1 or BARD1β was monitored over 3 days. BARD1β leads to increased proliferation, as described before. **e** Annexin V and PI staining of (**e**, **f**) A549 and (**e**, **g**) L929 24 h after transfection with FL BARD1, BARD1β or pcDNA control expressing plasmids. FL BARD1 induced cell apoptosis. Each bar represents mean + SD (*n* ≥2 experiments); two-tailed Student’s *T*-Test *p < 0.05, BARD1 versus control

Western blots and RT-PCR of cell extracts showed that fibronectin was upregulated in BARD1β overexpressing cells, as compared to FL BARD1 expressing or non-transfected cells (Fig. 3a, b), while no changes were observed for E-cadherin expression (data not shown). These findings might suggest that BARD1β affects fibronectin accumulation and cytoplasmic localization in A549 cells.

As previously reported for fibroblasts [23, 25], overexpression of BARD1β induced increased proliferation of A459 lung epithelial cells, which was not observed with FL BARD1 expressing cells or controls (Fig. 3c). Overexpression of FL BARD1 and BARD1β in the fibroblast cell line L929 showed an even more pronounced increase of proliferation for BARD1β, but proliferation arrest for FL BARD1 (Fig. 3d).

As FL BARD1 was known as a mediator of apoptosis [16–18], we tested whether FL BARD1 induced apoptosis in A549 and L929 cells, and to what extent. We stained control cells and FL BARD1 or BARD1β overexpressing cells for annexin v and analysed them by FACS (Fig. 3e-g). Only FL BARD1, but not BARD1β significantly induced apoptosis in both cell lines.

Together these results suggest that FL BARD1 and BARD1β can be upregulated by TGF-β, and increased BARD1β might promote cell proliferation and act in a pathway regulating stability and/or intracellular localization of fibronectin. FL BARD1 upregulation leads to increased apoptosis of epithelial cells and fibroblasts.

### BARD1 expression is upregulated in mouse model of lung fibrosis

Despite its limitations, several key features of human lung fibrosis are reproduced in the mouse model of lung fibrosis following administration of bleomycin [38]. These features include epithelial cell apoptosis and fibroblast proliferation and differentiation. We thus investigated whether BARD1 expression in lung tissues from mice with bleomycin induced lung fibrosis reflected the expression pattern observed *in vitro* and whether it paralleled the progression of the disease in this model. Importantly, this model permits to investigate BARD1 expression at early stages of the disease.

**Fig. 4:**
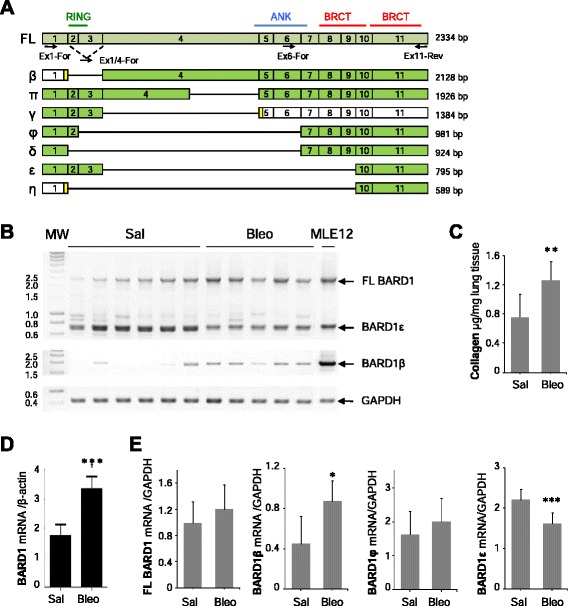
RNA expression pattern of BARD1 mRNA isoforms in bleomycin induced lung fibrosis. **a** Exon structures of mRNAs of FL BARD1 and isoforms are aligned. Locations of protein motifs are indicated as in Fig. 2a. Greek names of isoforms are indicated on the left and size in bp on the right. Exons with open reading frames (ORF) are marked as green, non-coding as white, alternative ORFs as yellow. Arrows indicate position of forward (For) and reversed (Rev) primers used for RT-PCR. **b** RT-PCR on lung tissues from control (Saline) and Bleomycin (Bleo)-treated mice at 15 days after treatment is shown, performed with primers amplifying exon 1 to 11 or the region from exon 1/4 junction (BARD1β-specific) to exon 11 (ex1/4- ex11). Amplicons of BARD1 isoforms are indicated with Greek letters. GAPDH was amplified as RNA quantity and quality control. **c** Collagen expression was determined by Sircol assay at 15 days after bleomycin treatment. **d** Quantitative PCR analysis at 15 days after bleomycin assay showed a significant increase of BARD1 expression. **e** Semi-quantitative RT-PCR analysis at 15 days after bleomycin treatment of FL BARD1, BARD1β, BARD1φ, and BARD1ε mRNA expression showed a significant increase of BARD1β expression and a highly significant decrease of BARD1ε. Each bar represents mean + SD (*n* ≥6 mice in each group); Student’s *T*-Test *p < 0.05, **p < 0.01, ***p < 0.005 Bleo- versus saline-treated

We investigated FL BARD1 and/or BARD1 isoform expression on the mRNA level by RT-PCR and determined which forms of BARD1 were expressed in lung tissues from bleomycin-treated and control mice (Fig. 4a, b). FL BARD1, BARD1β, and BARD1ε mRNA levels were the most abundant isoforms and BARD1β was significantly increased in the lungs of bleomycin-treated mice. To evaluate the overall extent of fibrosis, collagen deposition was measured in parallel using the sircol assay (Fig. 4c) and by measuring collagen type 1 alpha 1 levels by real time PCR (not shown). Real time PCR was similarly performed for expression of RNAs transcribed from exon 4 of BARD1 (Fig. 4d). To determine the expression of individual BARD1 isoforms we performed semi-quantitative PCR (Fig. 4e). The increase of BARD1β mRNA expression was statistically significant, but expression changes for FL BARD1 or other isoforms were not, with the exception of a significant down-regulation of BARD1ε. Whether BARD1ε plays a role, as protein or mRNA, in preventing lung fibrosis remains to be determined.

**Fig. 5:**
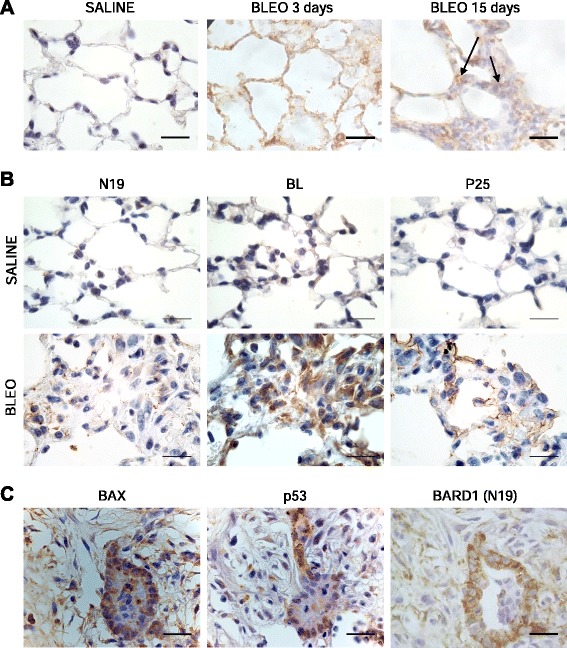
BARD1 epitopes differential expression and its association with apoptosis in in bleomycin-induced lung fibrosis in mice. **a** IHC of lung tissue of mice with bleomycin-induced lung fibrosis at 3 and 15 days after treatment (Bleo) and controls (Saline) using anti-BARD1 antibody (C20) directed against the BARD1 C-terminus. **b** IHC with antibodies against the N-terminal (N19) or middle region (BL) of BARD1, or the alternative ORF in exon 1 (P25) in saline or bleomycin-treated mice at 15 days after treatment. All antibodies show an increase of staining after bleomycin treatment. While N19 shows a dotted staining mostly in epithelial cells of alveolar walls, BL shows a strong staining of most cells both in alveolar area and dense fibrotic area 15 days after treatment. P25 staining, specific for BARD1β, is cytoplasmic and at the membrane of epithelial cells. **c** IHC on adjacent tissue sections using BARD1 N-terminal (N19), p53 and Bax antibodies is shown for dense fibrotic lung tissue. BARD1 is co-expressed with components of the BARD1-apoptosis pathway, p53 and Bax. Scale bars 20 μm

To analyze overall BARD1 expression in the mouse model of lung fibrosis, BARD1 C20 antibody recognizing a C-terminal epitope common to all isoforms was used. While only weak expression was seen in lung tissues of control mice, BARD1 expression was upregulated in lung tissues from bleomycin-treated mice (Fig. 5a). BARD1 expression was first observed in epithelial cells at 3 days following bleomycin treatment. At 15 days after treatment, the epithelium remained strongly positive, and fibroblasts in fibrotic regions also showed BARD1 expression, in both the nucleus and cytoplasm. To distinguish different forms of BARD1, we used antibody N19, specific for epitopes present in FL BARD1 and absent in BARD1β, which showed a faint dotted staining in cells in the fibrotic regions (Fig. 5b). The expression of epitopes present in FL BARD1 and BARD1β, recognized by antibody BL, was stronger and observed in most cells, presumably fibroblasts. The BARD1β-specific antibody P25 showed a similar staining as BL, suggesting that the staining observed with antibody BL mostly represents BARD1β staining (Fig. 5b).

These data show that BARD1 expression is modulated in the bleomycin model of lung fibrosis, and specifically BARD1β is upregulated in cells within fibrotic regions at 15 days post treatment.

### BARD1 expression is associated with apoptosis

As FL BARD1 mediates apoptosis by binding to and stabilizing p53, and BARD1-repressed cells are resistant to apoptosis [16], and apoptosis of epithelial cells is thought to be one of the mechanisms leading to epithelial cell loss in fibrosis, we investigated FL BARD1 expression and its association with apoptosis markers in fibrotic lung tissues of bleomycin treated mice. We observed that BARD1 staining in fibrotic regions coincided with the expression of proteins implicated in the BARD1-induced apoptosis pathway [16], namely p53 and Bax, (Fig. 5c). BARD1, p53, and the p53–induced pro-apoptotic protein Bax showed prominent staining in the epithelium adjacent to fibrotic areas that also stained for BARD1. These data are consistent with the previously described role of FL BARD1 in apoptosis [16–18].

### FL BARD1 and isoform BARD1β are expressed in human lung fibrosis

**Fig. 6:**
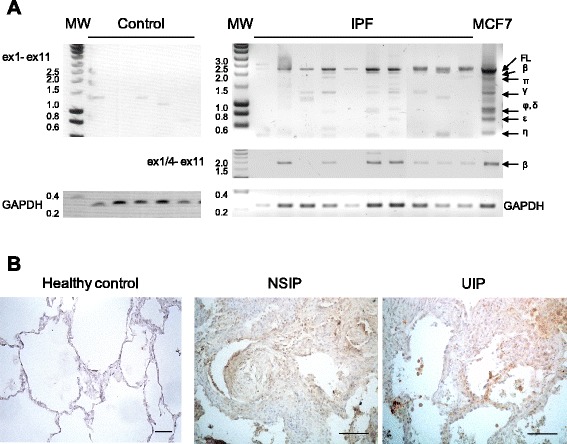
BARD1 expression is associated with human lung fibrosis. **a** RT-PCR with primers amplifying exon 1 to exon 11 (ex1- ex11) on tissues from control (non-symptomatic individuals) and a selection of IPF patients (total *n* = 17) is shown, performed with primers amplifying exon 1 to 11 or the region from exon 1/4 junction (BARD1β-specific) to exon 11 (ex1/4- ex11). Amplicons of BARD1 isoforms are indicated with Greek letters. GAPDH was amplified as control for RNA quality and quantity. In control lung tissues, only BARD1γ, BARD1δ, and BARD1η were detected, but no FL BARD1 or BARD1β. In tissues from fibrosis patients FL BARD1 and BARD1β were amplified in 70 percent of the samples. **b** Immunohistochemistry with anti-BARD1 antibody C20 shows representative cases of healthy lung tissue and tissues from patients with NSIP and UIP. Thickening interstitial areas and fibroblastic foci stained strongly for BARD1. Scale bars 100 μm

To evaluate the association of BARD1 with human lung fibrosis, we investigated its expression in lung biopsies from patients with pulmonary fibrosis (Fig. 6).

To determine the specific expression of FL BARD1 and/or BARD1 isoforms on the mRNA level, we performed RT-PCR on RNA extracted from lung biopsies from patients with pulmonary fibrosis (*n* = 17) and controls (Fig. 6a). Amplification of the entire BARD1 coding region (exon 1 to exon 11) showed no or very low expression of previously identified BARD1 isoforms in healthy tissues, but expression of FL BARD1 and BARD1β, as well as low expression levels of smaller isoforms was detected in IPF tissue samples (Fig. 6a). Using a BARD1β specific primer, we confirmed that BARD1β mRNA was the most frequently expressed in IPF samples.

We then performed IHC with antibody C20 recognizing a C-terminal epitope of BARD1 on tissue samples of NSIP as well as UIP patients. C20 showed BARD1 expression in NSIP and UIP, while only weak staining was observed in control tissues (Fig. 6b). BARD1 expression specifically localized to the fibrotic regions and was observed in all patients tested. The number of positive cells appeared to increase with progression of the fibrotic lesions, while minimal staining was observed in unaffected tissue. These data thus demonstrate that FL BARD1 and the spliced isoform BARD1β are expressed in fibrotic lung tissues and are associated with the disease.

**Fig. 7:**
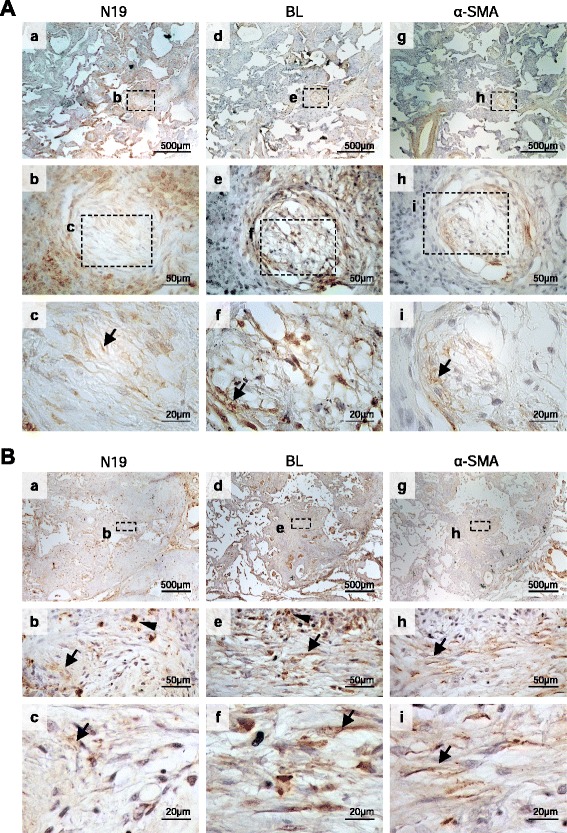
Differential expression of BARD1 epitopes in human lung fibrosis. **a** IHC of tissues of NSIP patients performed with anti-BARD1 N19 (**a**-**c**) and BL (**d**-**f**) antibodies. Adjacent tissue sections were also stained for α-SMA (**g**-**i**). Myofibroblast are preferentially stained by BL antibody and co-localized with α-SMA staining (*arrows*) in foci of loose proliferation of interstitial smooth muscle cells, as interstitial smooth muscle cells may be seen in the fibrosis pattern of NSIP. **b** IHC of tissues from IPF/UIP patients stained with anti-BARD1 N19 (**a**-**c**) and BL (**d**-**f**), and α-SMA (**g**-**i**) antibodies. Thickening interstitial areas stained strongly with N19 (arrow heads) (**b**, **c**). Regions that stained for α-SMA also were preferentially stained by BL (*arrows*) (**e**, **f**, **h**, **i**), suggesting co-expression of BARD1β and α-SMA in fibroblastic foci

IHC with antibodies directed against different epitopes of the FL BARD1 protein, namely N19 recognizing FL BARD1 and possibly small isoforms (Fig. 4a) and BL recognizing FL and BARD1β, showed that different epitopes of BARD1, reflecting different isoforms, were expressed in different regions: N19 detecting mainly FL BARD1 was highly expressed within the hyperplastic epithelium, but less in areas with collagen depositions and fibroblasts, as shown for NSIP and UIP (Fig. 7a, b). BARD1 BL staining, similar to P25 staining (Fig. 5b) and reflecting expression of BARD1β, was mostly observed in cells within fibrotic regions and coincided with α-smooth muscle actin staining. The same regions were less stained by N19, suggesting that isoform BARD1β and not FL BARD1 was expressed in fibrotic regions and presumably in fibroblasts.

## Discussion

We report here for the first time that the tumor suppressor BARD1 and its pro-proliferative isoform BARD1β are associated with pulmonary fibrosis and presumably involved in its pathogenesis.

### BARD1 a novel biomarker and possible treatment target for lung fibrosis

Several proteins, including NADPH oxidase 4, Toll-like receptor 3, CCL18, matrix metalloproteinase-7, and interleukin-8, besides TGF-β, are widely accepted as key players in pulmonary fibrosis [39]. In recent years several genes were identified that are critically involved in the pathogenesis of lung fibrosis [12, 40]. Some of the newly identified genes are involved in hitherto ignored pathways, namely cell-cell adhesion, host defense, and DNA repair [12].

The *BARD1* gene was not discovered as one associated with lung fibrosis in previously reported genetic screens. This could be due to the switching of the splicing pattern of the *BARD1* gene that is associated with lung fibrosis, which would need other approaches for detection than screening for mutations or overall expression changes. Indeed there is evidence that SNPs in *BARD1* that are associated with a subgroup of high-risk, aggressive, neuroblastoma, promote alternative splicing and expression of BARD1β [25, 41]. Exon and intron sequencing of the *BARD1* gene has identified mutations in exons and introns that affect splicing in breast and ovarian cancer [42]. It is therefore possible that SNPs in *BARD1* might be found associated with specific subclasses of lung fibrosis.

We hypothesized that the tumor suppressor BARD1, with functions in DNA repair pathways, plays a role in the pathogenesis of lung fibrosis, as previous reports suggested a role of BARD1 in the regulation of proliferation: i) BARD1 expression is upregulated in proliferating cells, ii) BARD1 isoforms are highly upregulated in cancer cells, and associated with uncontrolled or deregulated growth. As uncontrolled growth is also the feature of lung fibrosis, BARD1 expression was expected to be associated with lung fibrosis.

### Distinct expression patterns of BARD1 and its isoforms in lung fibrosis and cancer

The pro-proliferative functions of isoform BARD1β were first described in *in vitro* cultures of epithelial cells [23] and confirmed in non-transformed fibroblasts [25]. Consistent with its pro-proliferative action BARD1β was found expressed in most epithelial cell-derived cancers, but in combination with other isoforms and absence of FL BARD1 [13, 24, 26, 28]. In fibrotic lung tissues, the most significant expression was found for FL BARD1 and BARD1β. This difference is striking and might reflect the less complex nature of the cells forming the fibrotic tissue. Cancer cells have the capacity to evade apoptosis, grow independently of growth signals, have activated telomerase and unlimited growth, escape immune surveillance, and become invasive and metastatic [43]. Compared to these changes the proliferating fibroblasts have acquired only little cancer cell-like capacities. The activation of telomerase might be associated with lung fibrosis [44]. Fibrotic fibroblasts and epithelial cells express FL BARD1 and BARD1β. Loss of FL BARD1 leads to genetic instability, a hallmark of cancer cells, but not so much of lung fibrosis.

### A role of TGF-β in up-regulating FL BARD1 and its isoforms

**Fig. 8:**
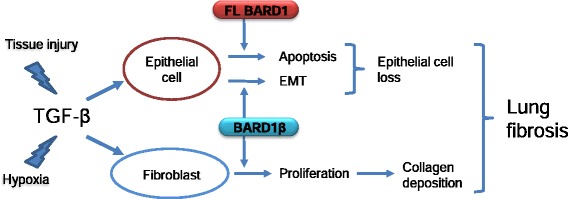
Signaling from TGF-β towards FL BARD1 and BARD1β expression in epithelial cells and fibroblasts might contribute to lung fibrosis

Knowing what causes FL BARD1 and BARD1β upregulation in fibrotic tissues may help us resolve whether the particular expression pattern is cause or consequence of the fibrotic transformation of lung tissue. Our data suggest that BARD1 and BARD1β are downstream of TGF-β in exerting effects on epithelial cells and fibroblasts (Fig. 8). Several reports have linked BARD1 expression to TGF-β-dependent pathways [33, 34]. Indeed, of the large number of transcription factors that specifically bind to the BARD1 promoter [45], several are activated in a TGF-β pathway (Additional file 1: Table S1), consistent with our finding that BARD1 expression can be regulated by TGF-β.

The data presented here confirm that TGF-β induces BARD1 expression and demonstrate for the first time that this occurs in lung fibrosis. Specifically, we observe upregulation of BARD1β on the mRNA and protein level in epithelial cells and fibroblasts. The upregulation of BARD1β in fibroblasts is consistent with the view that BARD1β acts as a pro-proliferative factor in a TGF-β pathway in lung fibrosis. Importantly, upregulation is cell type specific with marked differences between epithelial cells and fibroblasts. One explanation of these differences could be the differential expression of TGF-β receptors on epithelial cells and fibroblasts. It is also possible that other regulatory pathways are involved, such as microRNAs (miRNAs). Several miRNAs are implicated in TGF-β signaling, as recently reported for lung cancer [46]. The BARD1 3’UTR is target of a large number of miRNAs, including those induced by TGF-β signaling, that regulate the pattern of BARD1 isoform expression [45]. Further studies are required to unravel miRNA-mediated regulation of the TGF-β-dependent BARD1 expression.

*In vivo*, the fibrotic lung tissues from humans and mice showed an upregulation of FL BARD1 and BARD1β. *In vitro* experiments confirmed the induction of FL BARD1 and BARD1β on the transcriptional and protein level in response to TGF-β to some extent. *In vivo*, however, their expression might also be driven by hypoxia. Hypoxia is also a driver of miRNA expression in cancers and could play an additive role on the regulation of BARD1 expression, as BARD1 upregulation was observed in response to hypoxia *in vivo* [16, 20]. This could be reproduced in lung epithelial cells and fibroblast in culture (Additional file 1: Figure S1).

### Mechanism of action of FL BARD1 and its isoforms in epithelial cells and fibroblasts

The intracellular functions of FL BARD1 and its isoforms are not well understood. FL BARD1 is mostly bound to BRCA1, and the BRCA1-BARD1 heterodimer has E3 ubiquitin ligase activity [14, 15]. Several proteins have been discovered that are ubiquitinated and degraded by the BRCA1-BARD1 ubiquitin ligase [14, 47], including proteins that act in a signaling pathway from genotoxic stress to repair [48]. Proteins that play a role in cell cycle control, such as the centrosomal protein gamma-tubulin, and Aurora kinases, have also been identified as targets of the BRCA1-BARD1 E3 ligase [22, 23]. The isoforms lacking the BRCA1 interaction domain still retain the domain required for interaction with the ubiquitin ligase target protein. This is the case for BARD1β, which lacks the RING domain, but contains the Aurora kinase binding domain [23, 25]. BARD1β, by binding to Aurora A and B, antagonizes their degradation. Aurora kinases are required for progressing through cell cycle checkpoints and for completion of cytokinesis [49]. In non-transformed cells, Aurora kinases are synthesized during S-phase and gradually degraded during mitosis, in cancer cells their upregulation leads to uncontrolled progression through mitosis. This is also observed by FL BARD1 repression and/or BARD1β upregulation [23, 25]. Thus, BARD1β antagonizes the function of FL BARD1 and BRCA1 and acts pro-proliferative by overriding mitotic checkpoints, in epithelial cells and fibroblasts. The expression of BARD1β in fibrotic lung tissues is consistent with the view that BARD1β might drive fibroblast proliferation by its binding to Aurora kinases. It is possible that BARD1β also binds to other hitherto not identified target proteins of the FL BARD1-BRCA1 ubiquitin ligase.

Fibrotic fibroblasts produce excessive collagen, and FL BARD1 and/or other isoforms could be involved in lung fibrosis due to their ubiquitin ligase function which could contribute to the accumulation of collagen via (de)regulation of collagen or other extra cellular matrix protein production and/or turnover, which remains to be investigated.

### Hypothesis and future directions

TGF-β has long been proposed as a key molecule central to the pathogenesis of lung fibrosis [50]. However, it has thus far proved to be an intractable target largely due to the pleiotropic effects of this molecule, as well as its potential tumorigenic effects, and indirect approaches to modulate TGF-β signaling are considered [51, 52]. We suggest that BARD1 might act as downstream activator of epithelial cell apoptosis and fibroblast proliferation and therefore might present a strong candidate target molecule for the treatment of lung fibrosis and possibly other diseases in which fibro-proliferation is a feature.

## Additional file

Additional file 1: Table S1. Table S1 shows a list of transcription factors that bind to the BARD1 promoter sequence and are relevant to TGF-β signaling. **Figure S1.** BARD1 is overexpressed in response to hypoxia. Lung epithelial cells (human A549) and fibroblasts (mouse L929) were cultured in normal condition or in hypoxia, and cell extracts were prepared after 24 and 48 hours. Western Blot using anti-BARD1 BL (mapping exon 4). In hypoxia conditions, both BARD1 FL and BARD1β are upregulated in epithelial A549 cells. In fibroblasts L929, we can observe an upregulation of BARD1β at 48h whereas FL BARD1 is only weakly expressed. **Figure S2.** No staining observed in the absence of primary antibody. IHC was performed on adjacent tissue sections from NSIP patients without primary antibody (a-c) or with anti-BARD1 BL antibody (d-f). **Figure S3.** BARD1 expression in non-malignant and malignant cell lines. BARD1 expression is stronger in the murine immortalized but non-tumorigenic cell line derived from alveolar type II pneumocytes (E10) than in tumour cell lines (MLE12, murine tumorigenic transformed lung epithelial cells and A549, human adenocarcinomic alveolar basal epithelial cells). **Figure S4.** FL BARD1 and BARD1β overexpression in A549 cells. (A) Exon structures of mRNAs of FL BARD1 and BARD1β. Arrows indicate position of forward and reverse primers used for RT-PCR. (B) Epithelial cells (human lung cancer cells A549) were transfected with FL BARD1 (FL and FL2) or BARD1β (β ) expressing plasmids, or control plasmid (Co), and cell extracts were prepared after 48 hours. Reverse transcription and PCR was performed using primers from human exon1 to 11 of FL BARD1 (ex1-ex11) and from human exon 1/4 junction (BARD1β -specific) to exon 11of BARD1β (ex1/4-ex11). GAPDH PCR was performed as a control for cDNA quantity. Detailed Description of Materials and Methods. (PDF 1516 kb)
