# Supplementary material for: BARD1 mediates TGF-β signaling in pulmonary fibrosis
Source: Respir Res. 2015 Sep 29;16:118. doi: 10.1186/s12931-015-0278-3 (PMC4587901; doi:10.1186/s12931-015-0278-3)
Supplement: Additional file 1: Table S1. — Table S1 shows a list of transcription factors that bind to the BARD1 promoter sequence and are relevant to TGF-β signaling. Figure S1. BARD1 is overexpressed in response to hypoxia. Lung epithelial cells (human A549) and fibroblasts (mouse L929) were cultured in normal condition or in hypoxia, and cell extracts were prepared after 24 and 48 hours. Western Blot using anti-BARD1 BL (mapping exon 4). In hypoxia conditions, both BARD1 FL and BARD1β are upregulated in epithelial A549 cells. In fibroblasts L929, we can observe an upregulation of BARD1β at 48h whereas FL BARD1 is only weakly expressed. Figure S2. No staining observed in the absence of primary antibody. IHC was performed on adjacent tissue sections from NSIP patients without primary antibody (a-c) or with anti-BARD1 BL antibody (d-f). Figure S3. BARD1 expression in non-malignant and malignant cell lines. BARD1 expression is stronger in the murine immortalized but non-tumorigenic cell line derived from alveolar type II pneumocytes (E10) than in tumour cell lines (MLE12, murine tumorigenic transformed lung epithelial cells and A549, human adenocarcinomic alveolar basal epithelial cells). Figure S4. FL BARD1 and BARD1β overexpression in A549 cells. (A) Exon structures of mRNAs of FL BARD1 and BARD1β. Arrows indicate position of forward and reverse primers used for RT-PCR. (B) Epithelial cells (human lung cancer cells A549) were transfected with FL BARD1 (FL and FL2) or BARD1β (β ) expressing plasmids, or control plasmid (Co), and cell extracts were prepared after 48 hours. Reverse transcription and PCR was performed using primers from human exon1 to 11 of FL BARD1 (ex1-ex11) and from human exon 1/4 junction (BARD1β -specific) to exon 11of BARD1β (ex1/4-ex11). GAPDH PCR was performed as a control for cDNA quantity. Detailed Description of Materials and Methods. (PDF 1516 kb) [file 12931_2015_278_MOESM1_ESM.pdf]

BARD1 mediates TGF-β signalling in pulmonary fibrosis

Pierre-Alain André[1,2], Cecilia M Prêle[3,4], Sarah Vierkotten[5], Stéphanie Carnesecchi[6], Yves Donati[6], Rachel C. Chambers[7], Jean-Claude Pache[8], Bruno Crestani[9], Constance Barazzone[6], Melanie Konigshoff[5], Geoffrey J. Laurent[4], Irmgard Irminger-Finger[1,2,4*]

Supplementary Table S1.

Table S1 shows a list of transcription factors that bind to the *BARD1* promoter sequence [1] and are relevant to TGF-β signaling.

Transcription Factor	Comments
ATF2	Mediates TGF-β-induced transcriptional activation of MMP-2
BACH1	Expression induced by TGF-β. Regulates induction of chemokines and extracellular matrix degrading enzymes, inhibiting motility, invasiveness and EMT.
CEBPB	EGF-induced C/EBPβ participates in EMT by decreasing the expression of miR-203
CREB1	CREB phosphorylation induced by TGF-β1 treatment. Substrate for activated PKA,which activates
CTCF	TGF-β promotes complexes between Smad proteins and CTCF.
E2F1	Regulated by TGF-β. E2F1-Regulated MicroRNAs Impair TGF-β-Dependent Cell-Cycle Arrest and Apoptosis in Gastric Cancer.
E2F4	TGF-β induces a repressor complex containing Smad3, Smad4, E2F4 which link survivin to TGF-β-induced apoptosis and tumor progression.
EGR1	Expression induced by TGF-β and mediates stimulation of collagen gene expression.
FOS	Cooperates with Smad3 and Smad4 to mediate TGF-β-induced transcription. KRAS and YAP1 converge on FOS and activate a transcriptional program involved in regulating EMT.
FOXA1	Key factor in the loss of epithelial traits during EMT in lung cancer. Essential in providing chromatin access to TGF-β-activated Smad2 and Smad4 and their subsequent DNA binding.
HDAC2	PELP1/HDAC2/miR-200 regulatory network is important in EMT and metastasis of breast cancer.
IRF1	Expression repressed by TGF-β.
JUN	Mediates TGF-β-induced fibroblast activation and fibrosis in systemic sclerosis.
KDM5B	Histone demethylase JARID1B/KDM5B is a corepressor of TIEG1/KLF10
MBD4	DNA glycosylase TGF-β-mediated demethylation
MEF2A	TGF-β-activated Smad3 represses MEF2-dependent transcription in myogenic differentiation. Smad proteins function as co-modulators for MEF2 transcriptional regulatory proteins
MXI1	Expression induced by TGF-β1.
MYC	c-myc expression repressed by TGF-β
NFIC	Implicated in TGF-β signaling and extracellular matrix deposition. NF1-C2 counteracts EMT, motility, invasiveness, and tumor growth.
NRF1	Blocks the suppression of iNOS expression by TGF-β
PML	Expression induced by TGF-β. PML interacts with Smad2/3 and SARA (Smad anchor for receptor
RUNX3	Involved in TGF-β-dependent and -independent cell growth inhibition and apoptosis induction pathways
SAP30	SAP30L expression is induced by TGF-β
SP1	Required for TGF-β-induced mesenchymal transition and migration in pancreatic cancer cells by inducing early transcription of oncogenic Smad7 in pancreatic cancer cells.
SRF	Expression induced by TGF-β. Mediates TGF-β-induced pulmonary myofibroblast differentiation.
STAT3	Involved in TGF-β1–induced apoptosis and EMT.
TFAP2A	Knockdown of TFAP2A resulted in a overall alteration of TGF-β-induced transcription.
YY1	Inhibits TGF-β- and BMP-induced cell differentiation.

Supplementary Figures

BARD1 is overexpressed in response to hypoxia

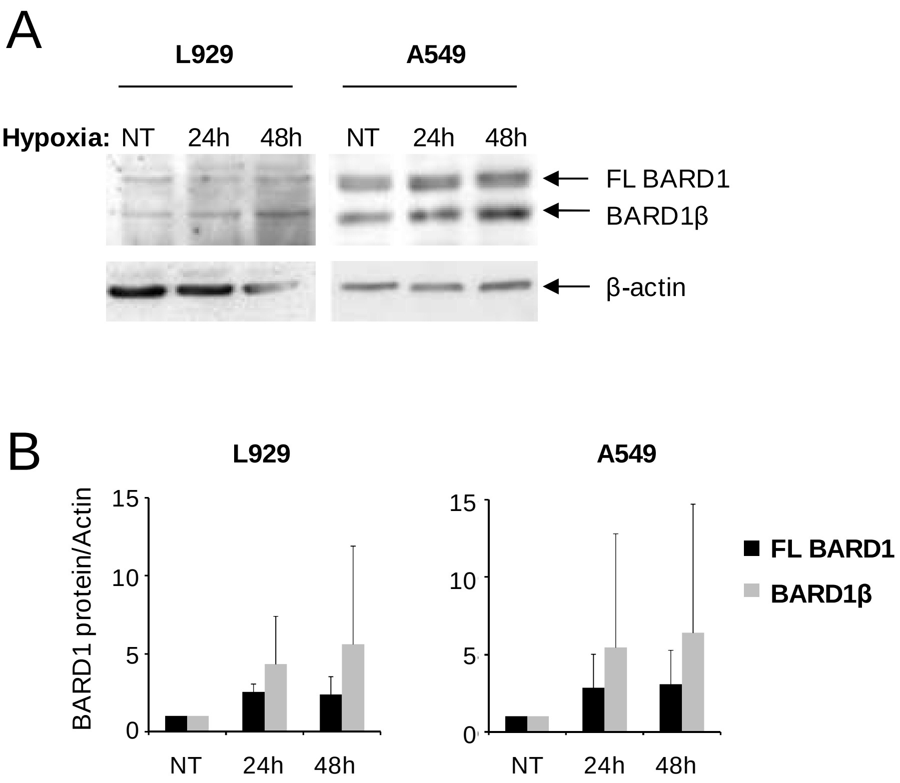

***Supplementary Figure S1*. BARD1 is overexpressed in response to hypoxia.**

Lung epithelial cells (human A549) and fibroblasts (mouse L929) were cultured in normal condition or in hypoxia, and cell extracts were prepared after 24 and 48 hours. Western Blot using anti-BARD1 BL (mapping exon 4). In hypoxia conditions, both BARD1 FL and BARD1β are upregulated in epithelial A549 cells. In fibroblasts L929, we can observe an upregulation of BARD1β at 48h whereas FL BARD1 is only weakly expressed.

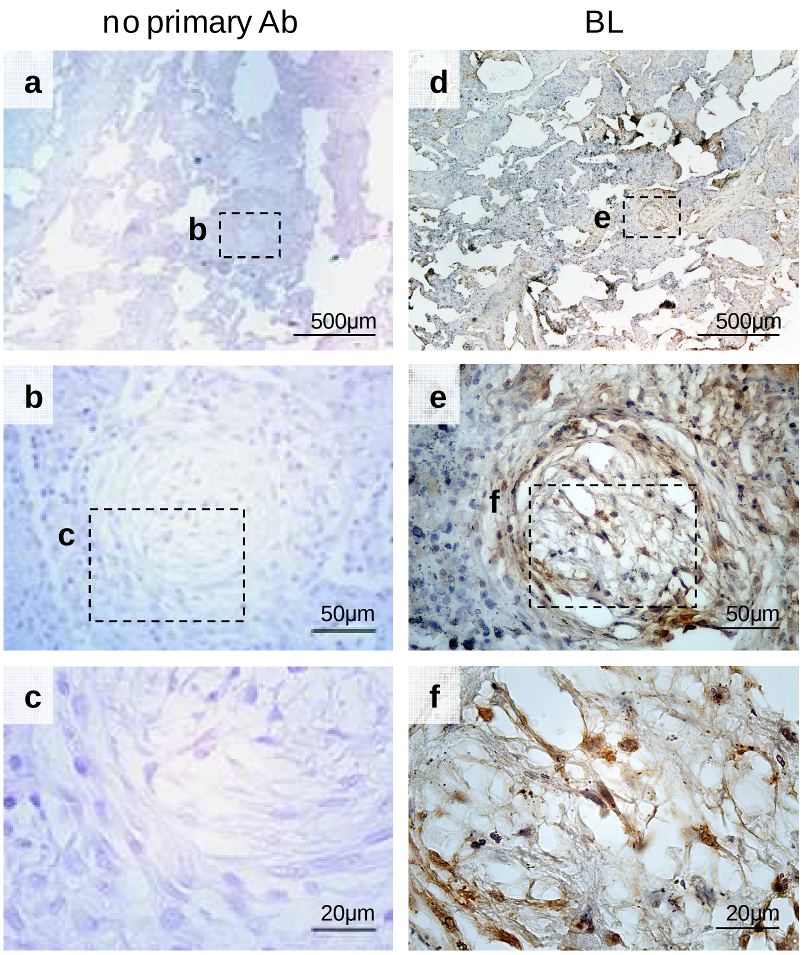

Supplementary Figure S2. **No staining observed in the absence of primary antibody.**

IHC was performed on adjacent tissue sections from NSIP patients without primary antibody (a-c) or with anti-BARD1 BL antibody (d-f).

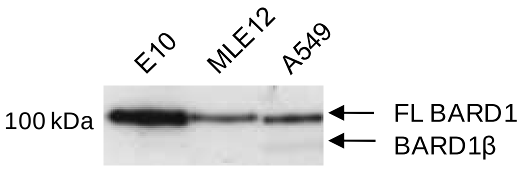

Supplementary Figure S3. BARD1 expression in non-malignant and malignant cell lines. BARD1 expression is stronger in the murine immortalized but non-tumorigenic cell line derived from alveolar type II pneumocytes (E10) than in tumour cell lines (MLE12, murine tumorigenic transformed lung epithelial cells and A549, human adenocarcinomic alveolar basal epithelial cells).

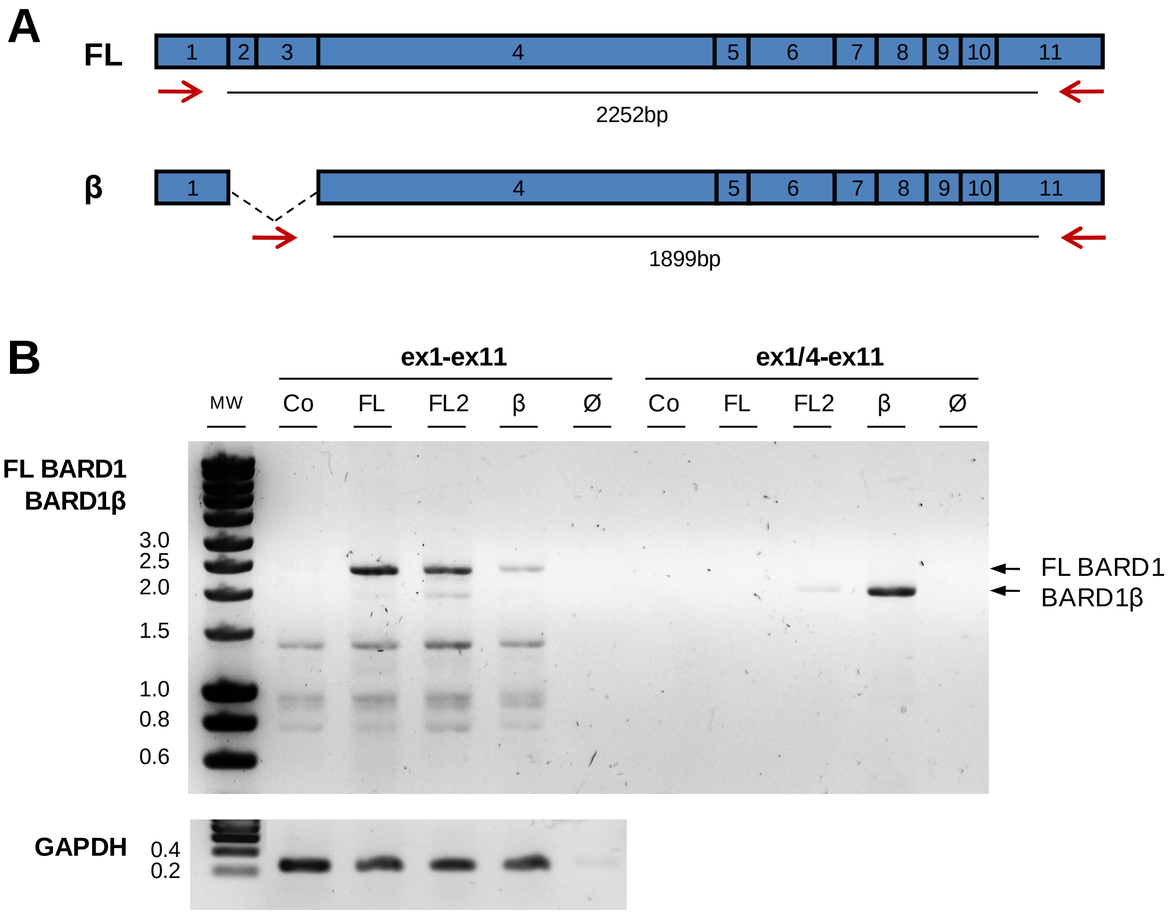

Supplementary Figure S4 . FL BARD1 and BARD1β overexpression in A549 cells. **(A)** Exon structures of mRNAs of FL BARD1 and BARD1β. Arrows indicate position of forward and reverse primers used for RT-PCR. **(B)** Epithelial cells (human lung cancer cells A549) were transfected with FL BARD1 (FL and FL2) or BARD1β (β) expressing plasmids, or control plasmid (Co), and cell extracts were prepared after 48 hours. Reverse transcription and PCR was performed using primers from human exon1 to 11 of FL BARD1 (ex1-ex11) and from human exon 1/4 junction (BARD1β-specific) to exon 11of BARD1β (ex1/4-ex11). GAPDH PCR was performed as a control for cDNA quantity..

Detailed Description of Materials and Methods

Sircol assay for collagen quantification

Total lung collagen content was measured in mouse lung tissue 15 days following bleomycin or saline treatment using the Sircol Collagen Assay Kit (Biocolor) in accordance with the manufacturer's guidelines. Briefly, lung samples were homogenized in 0.5M acetic acid containing 0.1 mg/ml pepsin. Pepsin soluble ECM collagen was then solubilised by a 24h incubation at +4°C. Insoluble fraction was separated by centrifugation. Clarified extracts were neutralized and treated with collagen isolation and concentration reagent by overnight incubation at +4°C. Sircol dye reagent (1ml), which specifically binds to collagen, was added to each sample and incubated for 30 min in a mechanical shaker. The collagen-dye complex was precipitated by centrifugation at 10,000 g for 10 min. The unfixed dye solution was carefully removed. After an acid-salt wash, the pellets were resuspended in 250µl of alkali reagent (0.5 M NaOH) to release the collagen bound dye in solution and placed in a 96-well flat-bottomed plate and absorbance at 570nm was measured. Collagen content was calculated by comparing sample values to a standard curve using cold acid-soluble bovine collagen Type I (0,5mg/ml) in 0,5 M acetic acid provided by the manufacturer as a standard.

Western blots

Cells were grown to confluence and stimulated for 24 or 48h with TGF-β1 with or without Smad inhibitor (SB431542). Cell cultures were placed on ice and washed 1x in ice cold PBS pH7.4. Cells were lysed in 50 µl of lysis buffer (10 mM Tris Base, 50 mM NaCl, 5 mM EDTA, 1 % Triton X-100 pH7.6) supplemented with 1 mM NaPyrophoshate, 2 mM NaOrthovanadate, 10 mM Na Molybdate, 5 mM Na Fluoride, 5 µg/ml Aprotinin, 5 µg/ml Leupeptin and 5 mM PMSF). Following 30 min incubation on ice and centrifugation at 11,000 rpm for 5 min at 4°C, the cleared cell lysates were collected and protein concentration was assessed by Bradford assays (Pierce). Approximately 15 ug of protein lysate was separated by SDS-PAGE, transferred onto PVDF membranes (Immobilon-P, Millipore, Bedford, MA, USA) and blocked with 5% (w/v) BSA in TBS-tween 0.05% (v/v) for 1h. Specific proteins were immuno-detected with antibody incubation over night at +4°C followed by incubation with HRP-conjugated secondary antibodies and visualized using enhanced chemiluminescence reagent (Amersham Biosciences) and X-ray film.

Membranes were probed for BARD1 BL, (A300-263A; Bethyl Laboratories; dilution 1:500) (exon 4), BARD1-p25 (alternative N-terminal region of BARD1β) [2, 3] (dilution 1:500), α-smooth-muscle-actin (α-SMA), (ab5694, Abcam, dilution 1:500), E-cadherin (sc-7870, Santa Cruz Biotechnology, dilution 1:500), Fibronectin (sc-6952, Santa Cruz Biotechnology, dilution 1:500). β-actin (A5316; Sigma; dilution 1:3000) or β-tubulin (sc-5274; Santa Cruz Biotechnology; dilution 1:1000) were used as a control for total protein loading.

Reverse Transcription-PCR

Total RNA was extracted from lung tissue (previously stored in RNAlater (Qiagen)) or from cell cultures using TRIzol reagent (Ambion) according to the manufacturer's protocol. cDNA was synthesized from approximately 1 µg of total RNA samples using either M-MLV Reverse Transcriptase and oligo(dT) (Promega) or omniscript reverse transcriptase (Qiagen) and random hexamer primers (Invitrogen Life Technologies) in accordance with the manufacturers guidelines. Semi-quantitative RT-PCR was performed on all genes analyzed. The cDNAs were mixed with deoxynucleotide triphosphate (dNTP), 10µM specific primers (Suppl. Table 1), Paq5000 HotStart DNA polymerase (Stratagene), and amplified with a PCR thermocycler. For all samples, a 5 minutes denaturation step at 95°C was followed by 35 cycles of amplification (30sec at 95°C, 30sec at 56°C, 30-180sec at 72°C) and 5 minutes of final elongation at 72°C. PCR products were separated on 0.8-1.2% agarose gels stained with ethidium bromide and visualized under UV light. GAPDH was amplified to 25 cycles and was used to determine equal concentrations of all cDNA gels. Primer sequences are shown in Supplementary Table 1.

For semi quantitative analysis, pictures of RT-PCR ethidium bromide agarose gels were acquired and band intensity was quantified using Fluorchem FC software (Alpha Innotech). Band intensity of mRNA of interest was expressed as relative to GAPDH to normalize the samples. Mean and standard deviation of all experiments performed were calculated after normalization.

Supplementary Table S2. Primer sequences for human and murine tissues and RT-PCR conditions. For all samples, a 5 minutes denaturation step at 95°C was followed by 25-35 cycles of amplification (30sec at 95°C, 30sec at annealing T°C, 30-180sec at 72°C) and 5 minutes of final elongation at 72°C.

Gene		Sequences (5'-3')	Annealing T°C	nb cycles
human FL BARD1	for	ATG CCG GAT AAT CGG CAG CC	57°C	35
	rev	CGA ACC CTC TCT GGG TGA TA		
human BARD1β	for	CTG CTC GCG TTG ATT TGA AAG	56°C	35
	rev	CGA ACC CTC TCT GGG TGA TA		
human GAPDH	for	AGC CAC ATC GCT CAG ACA CC	56°C	25
	rev	GTA CTC AGC GCC AGC ATC G		
murine FL BARD1	for	GAG GGT CTG CTC TGG GAA C	57°C	35
	rev	CGA ACC CTC TCT GGG TGA TA		
murine BARD1β	for	CTG CTC CCG CTG ATT CAA AAG	56°C	35
	rev	CGA ACC CTC TCT GGG TGA TA		
murine BARD1ϕ	for	CTG TAG TAA CAT ATT TGG TGT GC	56°C	35
	rev	CGA ACC CTC TCT GGG TGA TA		
murine BARD1ε	for	CAT GAC AAT AAA GGG GTG AAA G	56°C	35
	rev	CGA ACC CTC TCT GGG TGA TA		
murine GAPDH	for	TGT TCC TAC CCC CAA TGT GT	56°C	33
	rev	TGT GAG GGA GAT GCT CAG TG		

Real Time PCR

RNA were reverse transcribed using High Capacity cDNA Reverse Transcription Kit (Applied Biosystems, Foster City, CA). Real-Time PCR was performed in a 7300 Real-Time PCR System (Applied Biosystems). The amplification mixture contained 20 ng of cDNA, 5 µl 2x TaqMan Gene Expression PCR Master mix and 0.5 µl of specific 20x TaqMan Gene Expression Assay. Real-time PCR was performed with primers amplifying mouse BARD1 (AGACAACACATCTAGGGCAAGT and GGGAGTTGCGGAAACAAATTCAT). All samples were analyzed in triplicate and β-actin gene expression was used as housekeeping control (Applied Biosystems). The relative gene expression was calculated according the $2^{-\Delta\Delta CT}$ method.

Cell proliferation and apoptosis

Proliferation. 750'000 cells were seeded in 24-well plates and incubated overnight. Cells were then transfected with BARD1 expressing plasmids or control. The numbers of cells was counted at 0, 1, 2 and 3 days after transfection.

Apoptosis. Monitoring of apoptosis or necrosis was performed by staining with annexin V and PI (BD Pharmingen) according to the manufacturer's protocol. Briefly 250'000 cells/well were seeded in 12-well plates and incubated overnight. Cells were then transfected with BARD1 expressing plasmids or control and harvested 24 h after transfection. Cells were gently trypsinized, washed and resuspended in binding buffer. 100 µL of the cell suspension (100'000 cells) was transferred to a 5 mL culture tube, and a solution containing 5 µL FITC Annexin V plus 5 µL PI was added. The tube was gently vortexed and incubated for 15 min at room temperature in the dark. 400 µL of binding buffer was added, and the cells were analyzed by flow cytometry. The extent of apoptosis was determined as the percentage of Early (Annexin V+/PI-) and Late (Annexin V+/PI+) apoptotic cells. Flow cytometric analysis was performed with a BD Accuri C6 using BD Accuri software (BD, San Diego, CA, USA).

Immunofluorescence microscopy

Cells were fixed with 2% paraformaldehyde for 15 min at room temperature (RT). Fixed cells were permeabilized in 0.3% Triton/PBS for 15 min at RT and then blocked in 1% serum, 0.3% Triton/PBS for 30 min. Cells were incubated with appropriate primary antibodies overnight at +4°C in 1% FCS/PBS, washed, then secondary antibody for 1h at RT. Cells were then incubated in a DAPI solution before mounting using DAKO fluorescent mouting medium (S3023). Slides were analyzed under a Nikon epifluorescence microscope, and images were captured with a 3.3-megapixel CCD camera. Primary antibodies used were BARD1-BL (A300-263A; Bethyl Laboratories; dilution 1:50) (middle exon 4), E-cadherin (sc-7870, Santa Cruz Biotechnology,

Santa Cruz, CA; dilution 1:50), Fibronectin (sc-6952, Santa Cruz Biotechnology, Santa Cruz, CA; dilution 1:50).

References

1. Pilyugin M, Irminger-Finger I: Long non-coding RNA and microRNAs might act in regulating the expression of BARD1 mRNAs. Int J Biochem Cell Biol 2014, 54:356-367.

2. Bosse KR, Diskin SJ, Cole KA, Wood AC, Schnepp RW, Norris G, Nguyen le B, Jagannathan J, Laquaglia M, Winter C et al: Common variation at BARD1 results in the expression of an oncogenic isoform that influences neuroblastoma susceptibility and oncogenicity. Cancer Res 2012, 72(8):2068-2078.

3. Ryser S, Dizin E, Jefford CE, Delaval B, Gagos S, Christodoulidou A, Krause KH, Birnbaum D, Irminger-Finger I: Distinct roles of BARD1 isoforms in mitosis: full-length BARD1 mediates Aurora B degradation, cancer-associated BARD1beta scaffolds Aurora B and BRCA2. Cancer Res 2009, 69(3):1125-1134.
